# Effects of Microparticle Size and Fc Density on Macrophage Phagocytosis

**DOI:** 10.1371/journal.pone.0060989

**Published:** 2013-04-22

**Authors:** Patricia Pacheco, David White, Todd Sulchek

**Affiliations:** 1 George W. Woodruff School of Mechanical Engineering, Parker H. Petit Institute for Bioengineering and Bioscience, Georgia Institute of Technology, Atlanta, Georgia, United States of America; 2 Centers for Disease Control and Prevention Atlanta, Georgia, United States of America; University of Illinois, Urbana-Champaign, United States of America

## Abstract

Controlled induction of phagocytosis in macrophages offers the ability to therapeutically regulate the immune system as well as improve delivery of chemicals or biologicals for immune processing. Maximizing particle uptake by macrophages through Fc receptor-mediated phagocytosis could lead to new delivery mechanisms in drug or vaccine development. Fc ligand density and particle size were examined independently and in combination in order to optimize and tune the phagocytosis of opsonized microparticles. We show the internalization efficiency of small polystyrene particles (0.5 µm to 2 µm) is significantly affected by changes in Fc ligand density, while particles greater than 2 µm show little correlation between internalization and Fc density. We found that while macrophages can efficiently phagocytose a large number of smaller particles, the total volume of phagocytosed particles is maximized through the non-specific uptake of larger microparticles. Therefore, larger microparticles may be more efficient at delivering a greater therapeutic payload to macrophages, but smaller opsonized microparticles can deliver bio-active substances to a greater percentage of the macrophage population. This study is the first to treat as independent variables the physical and biological properties of Fc density and microparticle size that initiate macrophage phagocytosis. Defining the physical and biological parameters that affect phagocytosis efficiency will lead to improved methods of microparticle delivery to macrophages.

## Introduction

Uptake of particulate debris, fluid, and foreign substances by macrophages is a key aspect of the innate immune system [1], [2]. Macrophages are important generalist, first responder cells in the body that serve both recognition and degradative functions. Through recognition, engulfment, and processing of either self or non-self substances, macrophages remove waste; initiate, coordinate, regulate, and/or participate in immune responses; and monitor the body for deviations from homeostasis [3].

Biomedical applications that directly utilize phagocytosis stand to be substantially improved through greater understanding of the internalization process [4]–[6]. Particle internalization can be initiated through multiple pathways including toll-like receptors, scavenger receptors, complement receptors, chemokine or interleukin receptors, and the Fc receptor (FcR), which recognizes the crystallizable fragment of IgG antibody molecules [2]. Fc binding by macrophages initiates a number of signaling functions [7] that lead to actin-myosin driven phagocytosis [8], [9]. FcR-mediated phagocytosis of opsonized particles proceeds through both biomolecular and biophysical pathways that result in engulfment of the opsonized particle within a phagosome. After lysosome fusion to form a phagolysosome, oxidative, proteolytic, acidic, and other degradative processes decompose the engulfed substance [1], [2], [9]. The role of macrophages within the total immune response is broad, involving recruitment of many different cell types and interaction with cellular and molecular components to resolve the perceived “danger signal” [10]. For example, the Fc portions of immune complexes are also known activators for various components of the complement system, which then positively feeds-back to aid in the recruitment of additional macrophages [11]. Macrophages also assist in the progression from innate to adaptive immune responses. The ligation of Fc receptors decreases production of IL-12 [12], a cytokine key for the development of Type 1 helper T cell (Th1) phenotype [13], [14] while also driving T-cells into the Type 2 helper T cell (Th2) phenotype [14]. Th2 cell development subsequently leads to clonal expansion of B-cells and affinity maturation of produced antibody [15], aiding in the clearance of extracellular bacteria, viruses, and parasites [16].

Macrophages perform two important tasks through phagocytosis: sequestration and degradation of self particles (e.g. dead cells and debris), and elimination of foreign, non-self matter. In principle, both tasks proceed through a combination of physical cues, such as particle size, shape, and deformability [17], as well as biological cues such as recognition of pathogen-associated molecular patterns (PAMPs) or opsonized particles [2]. Therefore, it is likely that both physical and biological mechanisms are significant to regulating phagocytosis in macrophages. Understanding the biophysical and biological cues which trigger macrophage phagocytosis is important to improved utilization of phagocytosis in therapeutic microparticle delivery to macrophages.

Micro- and nanoparticles are commonly used and studied in the field of biomaterials, and specifically the study of phagocytosis, for applications such as drug delivery, vaccine delivery and development, and cancer therapies [18]–[21]. Microparticles have long been used to study phagocytosis [17], [22]–[25] in part due to their chemical and physical uniformity as well as their application in clinical settings. Multiple modeling studies on phagocytosis of particles, including computational models of 4–100 nm particles [26]–[28] and 3–11 µm particles [29]–[32], which include consideration of the effects of cell cytoskeleton and ligand density on phagocytosis. Experimental validation of these approaches which combine the effect of particle size and receptor density has been more limited. Previous experimental studies of Fc-mediated phagocytosis using microparticles [9], [23] did not examine the importance of the density of Fc ligands in conjunction with the size of the particle. Increasing the density of Fc on opsonized sheep erythrocytes caused macrophages to increase production of IL-10 and decrease production of IL-12 [33]. However in this study the effect of size and shape of the erythrocyte on phagocytosis was not explicitly examined or decoupled from Fc density. Other studies have shown that the shape of the particle can have a strong effect on phagocytosis [17]. In another study, the Fc density was varied on a spherical particle to understand the effects on cell signaling and commitment of the macrophage to phagocytosis. This study utilized 5.6 µm polystyrene particles with two different Fc densities and found that a high Fc density led to complete closure of the phagosome around the microparticle while a low Fc density resulted in binding of the particle to the macrophage but not internalization [23]. Numerous studies on phagocytosis have shown that particle size greatly affects the average number of microparticles internalized by the macrophage [17], [18], [25], but these did not address the impact of Fc density on internalization. Modeling and experimental approaches found that smaller particles are uptaken through both passive and active zipper mechanisms [29]. This study also found that actin polymerization is important for more regular phagocytic cups as it stabilizes receptor-ligand binding and suggests that active phagocytosis enables faster engulfment of the particle.

To evaluate the hypothesis that both Fc density and particle size significantly impact internalization by macrophages, we investigated the effects of Fc ligand surface density on phagocytosis for a variety of particle sizes. As macrophages may utilize different physical and biochemical mechanisms for clearing foreign objects of a wide range of sizes (submicron to greater than 5 µm), we explicitly decoupled the effects of the physical signal resulting from particle size and the biological signal resulting from variation in Fc density. By decoupling the biological and physical signals, we can better optimize particle-mediated delivery of therapeutic payloads to macrophages.

## Methods

### Microparticle Opsonization

**Figure 1:**
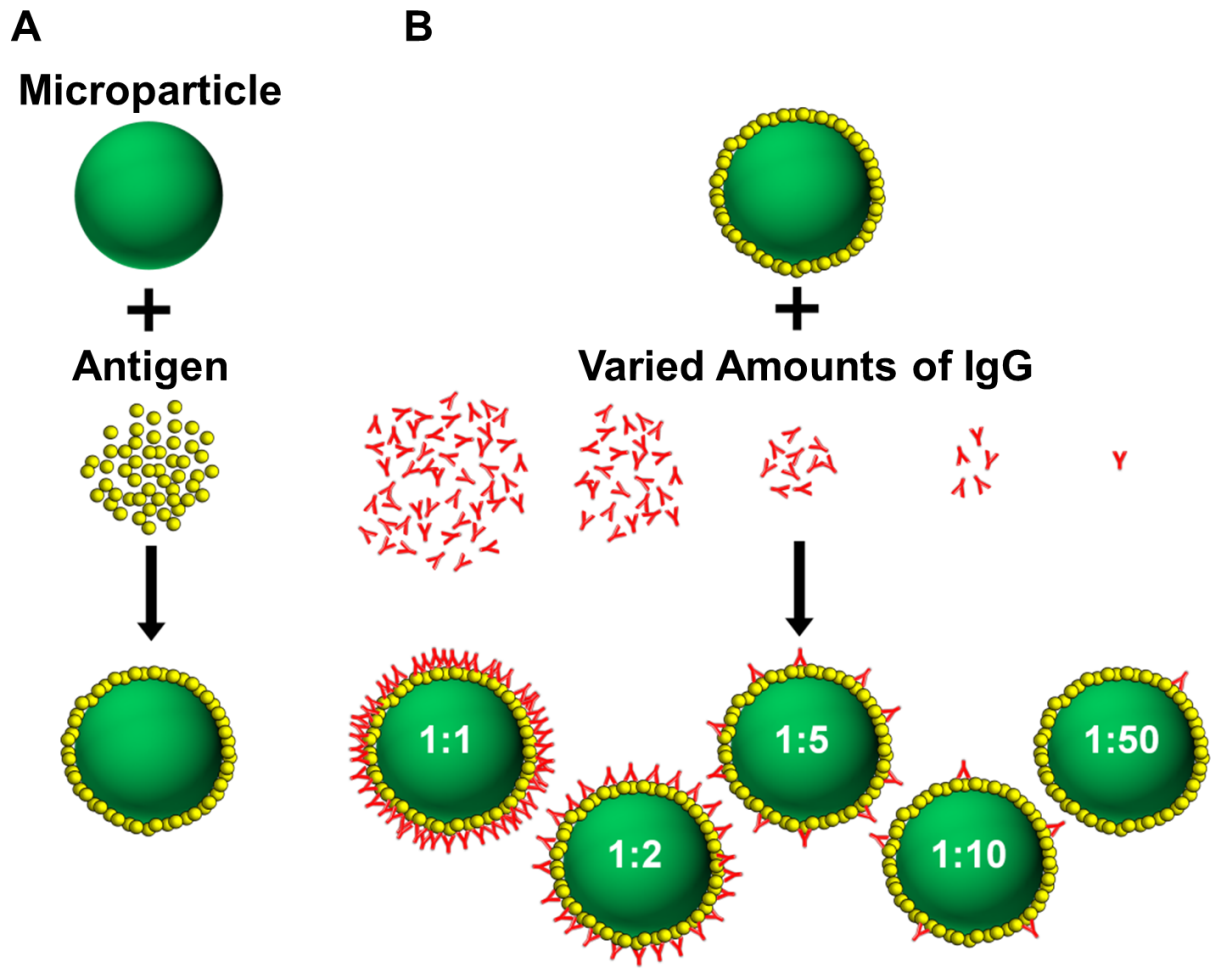
Microparticle opsonization. (A) Carboxylated polystyrene microparticles are first incubated with a BSA solution to ensure the BSA antigen is adsorbed onto the microparticle surface. (B) Varying amounts of sheep anti-BSA IgG was then added corresponding to a specific ratio of IgG to antigen. This process ensures external presentation of the Fc portion of the antibody molecule and was repeated for each microparticle size.

Carboxylated yellow-green fluorescent polystyrene microparticles were purchased from Polysciences (Warrington, PA). To examine the effects of a broad range of sizes on internalization efficiency, we used 0.5 µm, 1 µm, 2 µm, 3 µm, and 4.5 µm particles. A schematic showing the Fc opsonization process is shown in Figure 1. The microparticles were incubated in a 1 mg/mL bovine serum albumin (BSA) (Sigma Aldrich, St. Louis, MO) solution to adsorb a layer of antigen onto the particle surface. The concentration of BSA was chosen to be 10 times the saturating concentration for the amount of beads, as determined from manufacturer's specifications. The concentration of BSA was increased in direct proportion to the total microparticle surface area to remove the density of the antigen as an experimental variable. To create particles with different densities of exposed Fc domains (i.e. different valencies), sheep polyclonal anti-BSA IgG antibody (Abcam, Cambridge, MA) was added in decreasing antibody mass to BSA mass ratios thereby producing a range of exposed Fc densities, or Fc density ratio. We identified a 1∶1 ratio of added antibody to antigen that represents a condition of maximum Fc coverage. Dilutions of 1∶2, 1∶5, 1∶10, and 1∶50 ratio of added antibody to antigen were tested. No added IgG, or a 0∶1 ratio, indicates microparticles coated only with BSA antigen. A table of the amount of BSA antigen and opsonizing antibody added to each particle size is shown in Figure S2. Microparticles were incubated with protein at room temperature for at least 2 hours in phosphate buffered saline (PBS) at a pH∼7.2 (Invitrogen, Carlsbad, CA) for all protein-binding steps. After each binding step, the microparticles were washed three times by centrifugation and PBS exchange. We observed only slight particle clustering in the case of 0.5 µm particles and negligible clustering for particles of other sizes (data not shown). To verify the availability of Fc domains and to quantify the amount of Fc presented on each microparticle, a 10 µL aliquot from each Fc density condition was incubated with a TexasRed fluorescently labeled rabbit anti-sheep IgG secondary antibody (Abcam, Cambridge, MA). This process was repeated to create duplicate particle groups.

### Cell Culture

RAW 264.7 murine macrophages were used in the internalization assay. These cells were a gift from the lab of Niren Murthy and were originally purchased from ATCC (Manassas, VA). The cells were cultured in complete media composed of DMEM (Sigma Aldrich, St. Louis, MO) supplemented with 10% fetal bovine serum (Atlanta Biologicals, Atlanta, GA) and 0.1% penicillin/streptomycin, and were grown in a humidified incubator at 37°C with 5% CO_2_. For the internalization assay, cells were plated in a standard 6-well tissue culture plate at a density of approximately 3×10^5^ cells per well. The cells were incubated in complete media for 36–48 hours until fully adherent. Supernatants were aspirated and the plates were washed twice with PBS. After the final aspiration, 0.5 mL of fresh DMEM, followed by the microparticles, was added to each well.

### Internalization Assay

Approximately 25 microparticles per cell were added to each well of plated macrophage cells for each condition tested. The cells and microparticles were gently mixed and then allowed to interact in a humidified incubator at 37°C with 5% CO_2_ for approximately 1½ hours. A duplicate set of plated samples were incubated at 4°C to determine the rate of microparticle adhesion. After the incubation period, the well contents were aspirated and washed with PBS twice to remove particles that did not interact with the adherent cells. 1 mL of PBS was added after the final washing was complete. The well was then scraped using a standard cell scraper and the entire contents were transferred to a microcentrifuge tube for analysis using flow cytometry. The internalization assay is illustrated in Figure S1 and was repeated 4 times for each Fc density and size condition.

### Flow Cytometry and Microscopy

All samples were analyzed using a flow cytometer (Accuri C6) immediately after microparticle-cell incubation, collection, and mixing. To measure the amount of TexasRed-labeled secondary antibody per microparticle, the 630±30 nm bandpass filter (suitable to detect TexasRed) was used in conjunction with the 533±30 nm bandpass filter to detect the yellow-green fluorescence of the microparticle core. As the TexasRed emission spectrum overlaps part of the yellow-green fluorescence of the particles, results were compensated by subtracting the overlapping signal of the secondary antibody from that of the particle using the compensation tool in the CFlowPlus software (BD, Franklin Lakes, NJ). Due to high levels of overlapping fluorescence from the particle and secondary antibody, non-fluorescent microparticles and a FITC-labeled secondary antibody were used to estimate the density of exposed Fc on non-fluorescent particles. Flow cytometry was also used to estimate the particle concentration for each condition. To determine the number of microparticles phagocytosed for each Fc density condition and particle size, the populations were analyzed using forward scatter and microparticle core fluorescence. Events were gated on forward scatter to separate the larger cells from the smaller microparticles. Three populations were delineated: macrophage cells alone, microparticles alone, and microparticles collocated with cells. The number of microparticles per cell was then determined by measuring the mean fluorescent intensity (MFI) of the dual-signal population and dividing it by the average MFI of one particle within the dually-positive population, in accordance with previous methods [34]. ANOVA followed by Tukey-Kramer HSD post analysis was performed to determine significance using JMP software (SAS, Cary, NC).

In preparation for fluorescence microscopy and confocal microscopy, cells were plated onto sterile coverslips within a 6-well plate before the internalization assay. Each well was then washed with PBS 3 times and 0.5 mL of PBS added to each well after the final washing. The cells were stained in a 5 µg/mL solution of a lipid specific cell membrane stain, FM4-64X (Invitrogen, Carlsbad, CA) for 1 minute. After the incubation period, the cells were fixed with a 4% paraformaldehyde solution for 10 minutes and then rinsed 3–5 times with PBS. The cells were then treated with 4′,6-diamidino-2-phenylindole (DAPI) to stain nuclear DNA. After a short incubation period, the coverslips were then removed from the well and transferred to a standard microscope slide. The stained cells were visualized with a Nikon Ti epi-fluorescence microscope and Zeiss LSM 510 VIS confocal microscope. Images were processed with the Nikon Elements and Zen Lite 2011 software.

## Results

### Fc density increases with antibody to antigen ratio

**Figure 2:**
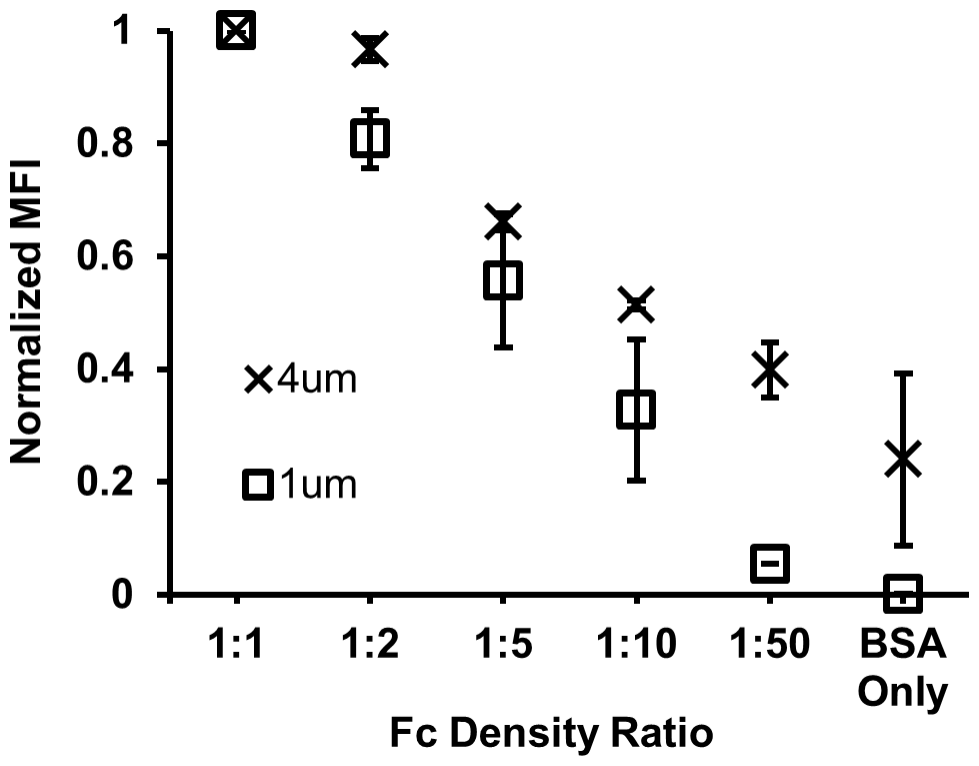
Normalized mean fluorescence intensity of microparticles labeled with fluorescently conjugated secondary antibodies. Decreasing normalized MFI corresponds to decreased Fc density for fluorescent particles.

The Fc density was estimated for each surface dilution and particle size using the MFI of the secondary antibody signal that was normalized to the maximum saturated value. The normalized MFI decreased as the Fc was diluted both for the larger particles (4 µm) and smaller particles (1 µm). (Figure 2) These data indicate that Fc density was successfully manipulated for each particle size. Similar trends in Fc density were observed by flow cytometry for the fluorescent particles of all sizes used in the internalization assays (Figure S3).

### Effects of Fc density on microparticle adhesion to cell surface

**Figure 3:**
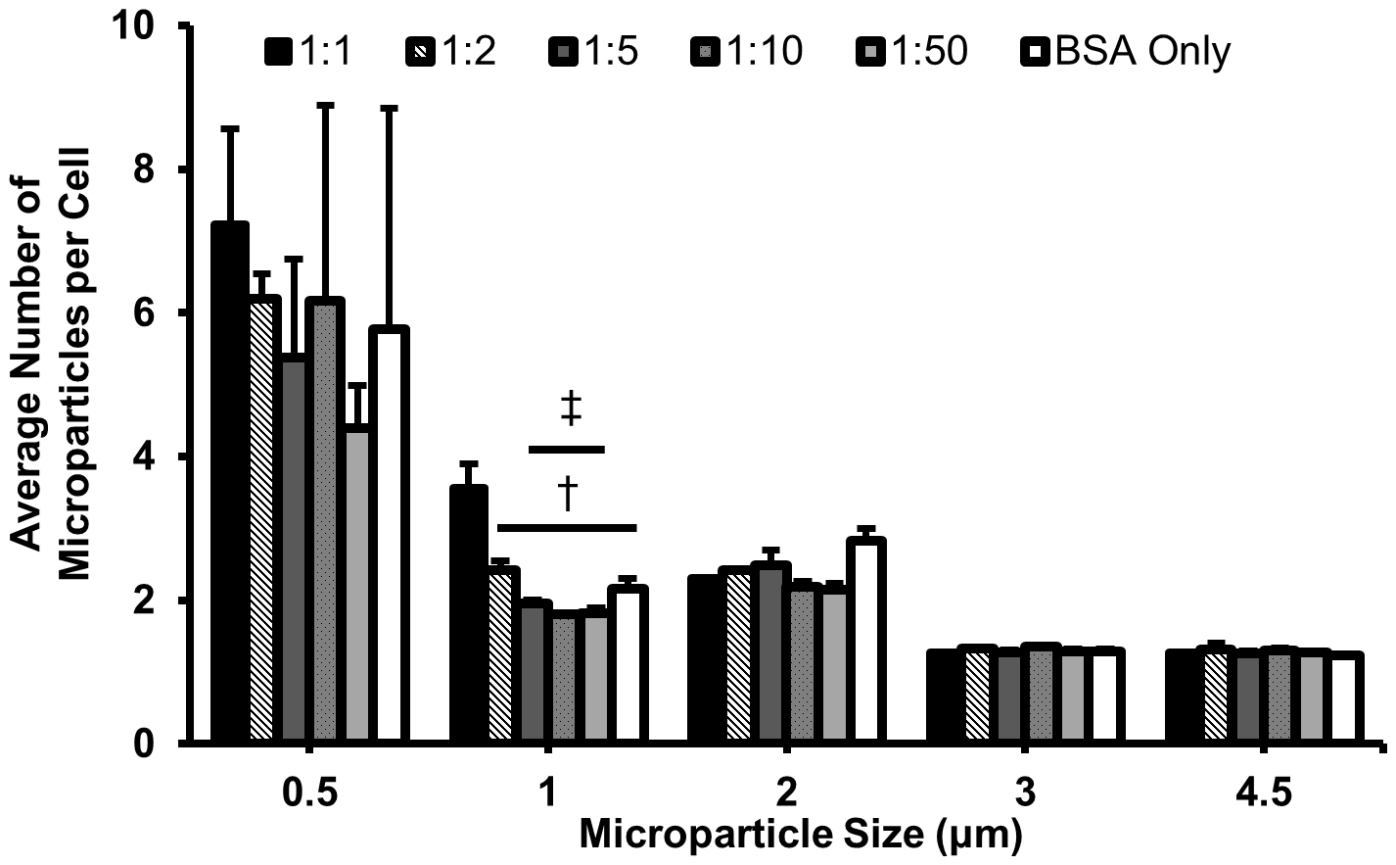
Average number of attached microparticles for each particle size and Fc density condition. As particle size decreases, so does the attachment efficiency. Overall, Fc density was not a significant variable for opsonized and BSA-only particles. †- Significantly different (SD) from 1∶1, ‡- SD from 1∶2, with p<0.05, (N = 2).

To determine the influence of Fc density and particle size on microparticle adhesion to macrophages, we performed the same internalization assay at 4°C, a temperature which inhibits internalization but not attachment [24], [25]. The average number of microparticles attached per cell was estimated from flow cytometric analysis for each Fc density and particle size condition (Figure 3). ANOVA was conducted to examine the independent effect of Fc density and particle size on cell attachment. Attachment of the 0.5 µm particles was significantly greater than that for the other four sizes. High Fc density also increased attachment of the 1 µm particles, but it did not enhance adhesion for the other particle sizes. Therefore, Fc does not play a strong role in particle attachment, with the exception of 1 µm particles at high density of Fc. However, the lack of cellular activity at this temperature (active engulfment, receptor recruitment, etc.) may alter the adhesive properties at physiological conditions.

### Effects of Fc density on microparticle internalization

**Figure 4:**
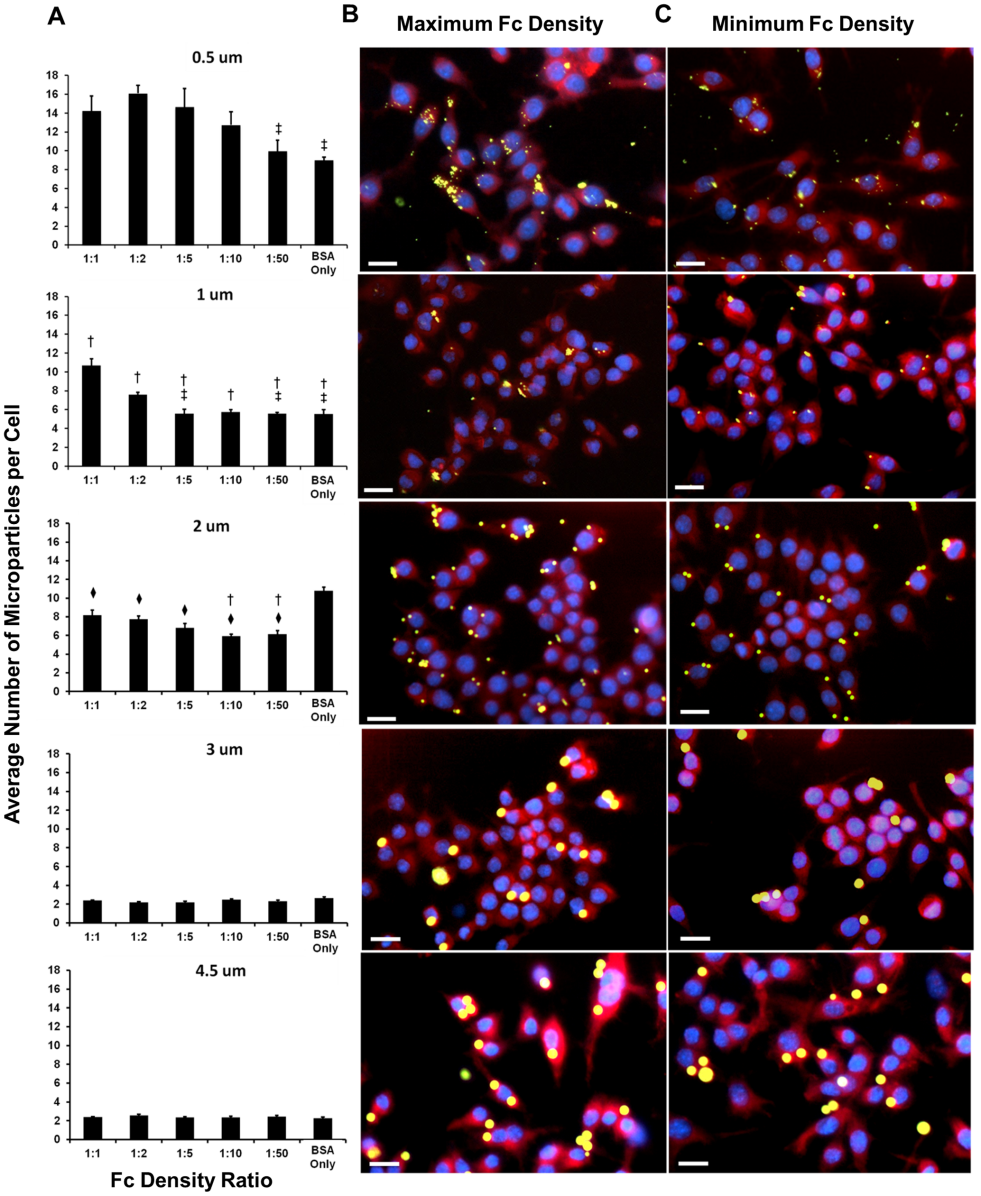
Average number of microparticles internalized per cell for each particle size and Fc density condition. (A) Average number of microparticles per cell for each particle size for the various Fc density conditions. (†- Significantly different (SD) from 1∶1, ‡- SD from 1∶2, ♦- SD from BSA Only, with p<0.05). These values were also illustrated through fluorescent microscopy by comparing images taken of RAW264.7 macrophages incubated with Fc functionalized particles. (B) Representative images for each size with the maximum Fc density ratio of 1∶1. (C) Representative images for each size with the minimum Fc density ratio of 1∶50. Scale bar represents 20 µm. (N = 4).

The average number of microparticles internalized per cell was measured with an internalization assay at 37 °C (Figure 4a). ANOVA of the average number of microparticles internalized per cell showed that both size and Fc density are significant variables at physiological temperatures. For smaller and medium-sized particles (0.5 µm, 1 µm, and 2 µm), the number of microparticles internalized per cell was significantly greater for the higher Fc density conditions of 1∶1 and 1∶2 than the lower Fc density conditions of 1∶10 and 1∶50. There was a significant decrease in the average number of microparticles internalized per cell for the 3 µm and 4.5 µm particles. Fc density was not a significant factor in the internalization of larger 3 µm and 4.5 µm particles. In addition, larger particles (3 µm and 4.5 µm) were generally internalized at lower numbers than small and medium-sized particles (0.5 µm, 1 µm, and 2 µm), for a given Fc condition. However it should be noted that the medium-sized 2 µm particles showed an increase in internalization of BSA-only coated samples.

Representative images of the macrophages and microparticles after incubation are shown for the high Fc density ratio of 1∶1 and the low Fc density ratio of 1∶50 (Figure 4b, c). Inspection of these images illustrate the extent to which internalized microparticles are not distributed equally among macrophages, but instead tend to be phagocytosed by a subset of cells, especially for smaller particles functionalized with a high Fc density.

**Figure 5:**
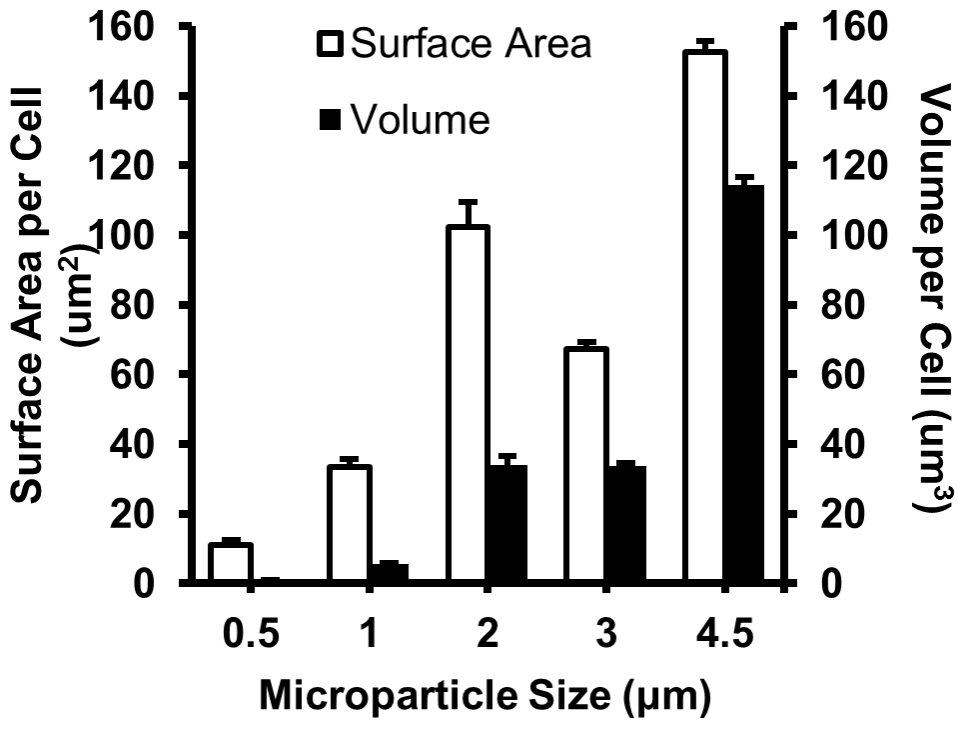
Average total volume and surface area of internalized microparticles. Average calculated volume and surface area of microparticles internalized for the maximum Fc condition of 1∶1 for each particle size. (N = 4) The results for each particle size are significantly different from each other (p<0.01).

While the average number of phagocytosed microparticles per cell was significantly reduced for large particle sizes, the total volume of phagocytosed particles per cell increased with particle size. The total surface area of the internalized particles was also maximal for the 4.5 µm particles (Figure 5).

### Combined effects of Fc density and microparticle size on phagocytic activity of macrophages

**Figure 6:**
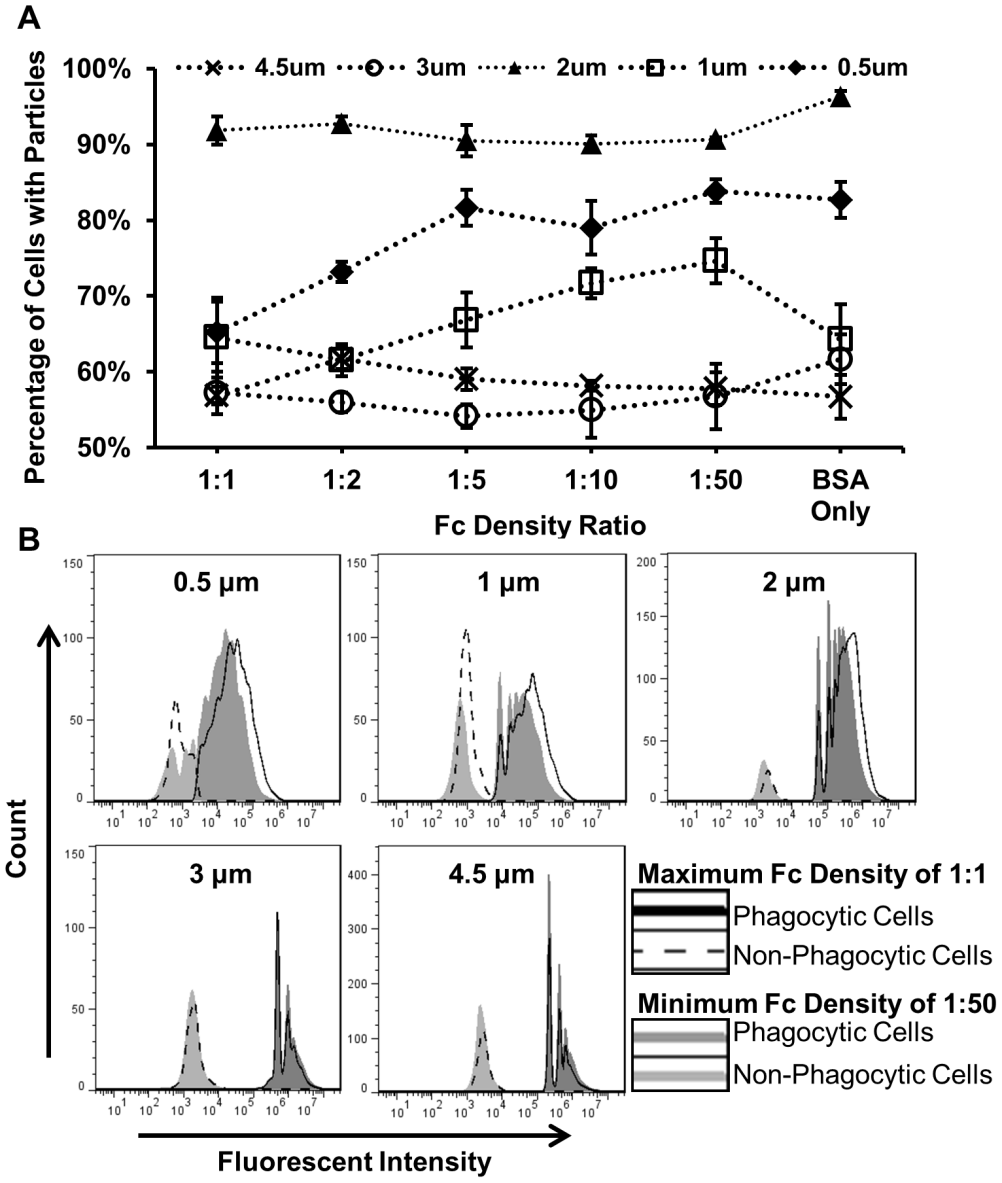
Effects of Fc density and particle size on the macrophage population. (**A**) Percentage of phagocytic cells at each Fc density condition for each particle size with a dashed line to illustrate trends. (N = 4) (**B**) Representative histograms of the FITC intensity for macrophages incubated with particles functionalized with the maximum Fc density of 1∶1 and the minimum Fc density of 1∶50. These histograms illustrate two groups: macrophages that have internalized at least one particle (phagocytic cells) and macrophages that did not internalize a particle (non-phagocytic cells).

The previous data show the decoupled effects of particle size and Fc density on the number of internalized microparticles. Since the number of macrophages plated was constant for each tested condition, we were able to compare the percentage of total macrophages that were phagocytic for each treatment using flow cytometry data. We considered a macrophage to be phagocytic if it contained at least one microparticle. The percentage of phagocytic macrophages was found to be strongly dependent on both the particle size and the particle Fc density (Figure 6a). Interaction with the smaller particles (0.5 µm and 1 µm) at a low Fc density resulted in a greater percentage of phagocytic macrophages than with high Fc density. In contrast, the larger particles (3 µm and 4.5 µm) resulted in a slightly greater percentage of phagocytic macrophages for the high Fc density condition. For the 2 µm particle treated cells, the average percentage of phagocytic macrophages remained relatively constant regardless of Fc density, and was consistently higher compared to the other particle sizes.

Combining the results of macrophage phagocytic activity with the average number of particles internalized shows that for small particles (0.5 and 1 µm) a smaller percentage of the macrophages may be phagocytic, but they internalize a relatively large number of particles. The shift to a higher fluorescence signal for the high Fc density condition, observable for the 0.5 µm, 1 µm, and 2 µm particles, indicates that an increased average number of particles were internalized by phagocytic cells. In addition, there is an increase in the number of non-phagocytic cells seen in the 0.5 µm and 1 µm high Fc density histograms compared to the low Fc density, as indicated by an increased count of low fluorescence events. (Figure 6b). There was no observable shift in fluorescence dependent on Fc density for the larger particles of 3 µm and 4.5 µm. However this was expected as Fc density was a not a significant variable for the larger particles in our internalization studies. These data support the hypothesis that while the smallest particles (0.5 µm and 1 µm) functionalized with the highest Fc density are internalized in greater numbers, a smaller number of macrophages are involved in the internalization process.

## Discussion

One important mechanism for recognizing threats in the body is opsonization—the coating of invading pathogens with antibodies marks them as a “threat” or a “danger signal”. This process can prevent biological activity of pathogenic molecules through steric interference (e.g. interfering with binding to cells or other biological targets), but also tags the pathogen for recognition by macrophages and other antigen-presenting cells through the external orientation of the constant region of the antibody molecule known as the crystallizable fragment (Fc) [35]. In these studies we quantified the phagocytic activity of macrophages as a function of Fc density and particle size.

**Figure 7:**
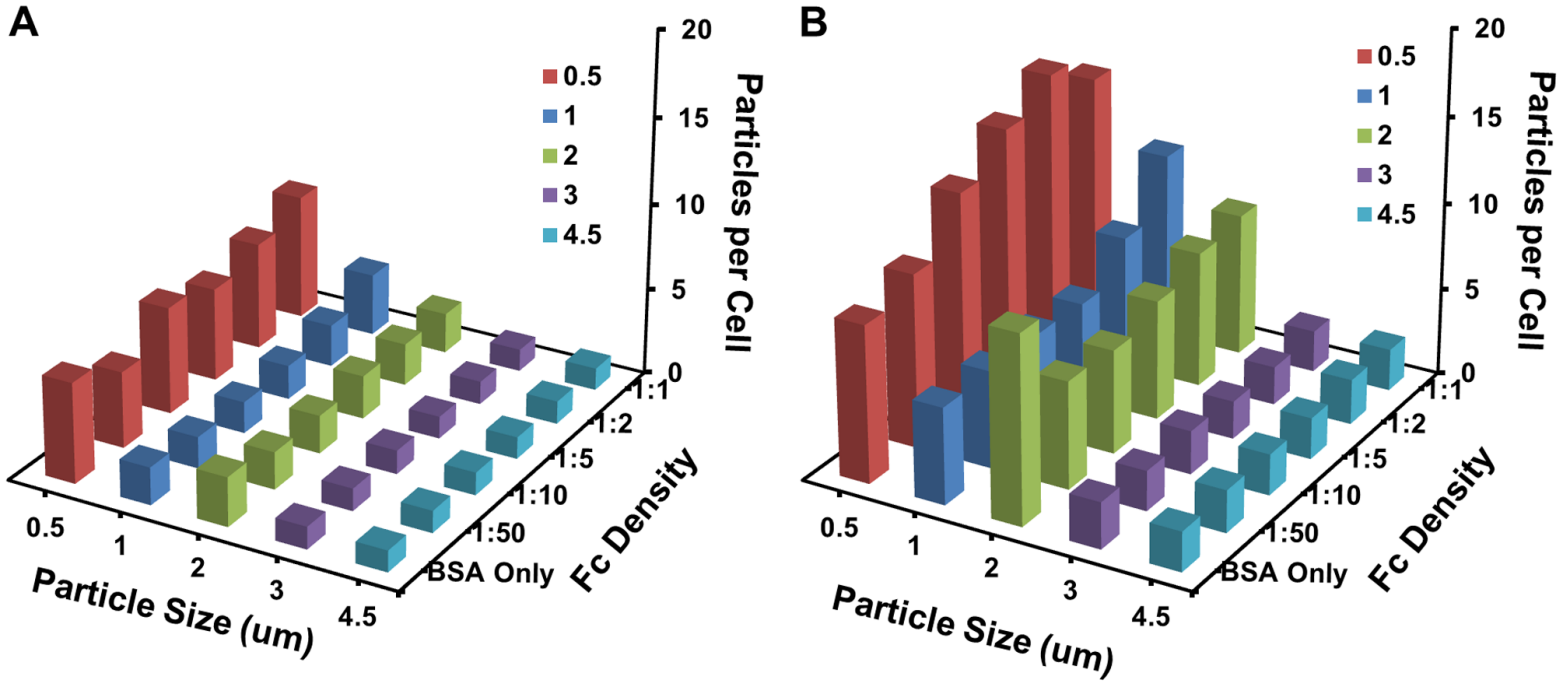
Summary of the average number of attached and internalized microparticles per cell for each particle size and Fc density condition. (A) The attachment of particles to macrophages does not have a strong dependence on microparticles size and Fc density. (B) The internalization of particles by macrophages is highly dependent on microparticle size and Fc density with an overall trend of the average number of internalized particles decreasing as microparticle size increases and Fc density decreases.

We successfully varied the Fc density on microparticles for all tested sizes. We then analyzed the effects of Fc density on attachment alone by conducting a low temperature study. We found that while particle size did play a role in attachment, Fc density did not. The results of our particle internalization assays showed a strong dependence between the Fc density and particle size on the average number of microparticles adhered to and internalized by the macrophage population (Figure 7 and Figures S4 and S5). While size dependence of particles has been shown to affect phagocytosis in previous studies [25], [36], [37], this is the first study to look at the combined effects of Fc density and particle size on phagocytosis, which both play an important role [26]. Smaller particles (0.5 µm and 1 µm) showed a strong correlation between Fc density and the average number of internalized microparticles per cell. However for the larger particles (3 µm and 4.5 µm), an increase in Fc density resulted in no significant change in the average number of microparticles phagocytosed per cell. For the 2 µm BSA-only coated particles, an apparent anomaly from the general trend was seen in the average number of internalized particles per cell. The BSA-only particles were internalized in significantly higher levels compared to the Fc-functionalized particles. Others have shown that BSA-only particles can be internalized by macrophages, most likely through scavenger receptors [38], [39]. However, the 2 µm particle may represent a special case due to the topography of the macrophage membrane. Previous studies have shown that the macrophage membrane ruffles are specially tuned with an average curvature of approximately 2 µm [25].

The fluorescent microscopy images of the internalization of small particles (0.5 µm and 1 µm) (Figure 4b, c) showed a great disparity in the phagocytic activity of individual macrophages, which we confirmed through further flow cytometry analysis (Figure 6). High density of Fc resulted in an increase in phagocytosed particles, but also an increase in cells that do not phagocytose any particles (Figure 6b, 0.5 µm and 1 µm). Since our macrophage line is assumed to be relatively homogenous, the enhanced activity seen only when small particles were added, could be due to a positive feedback mechanism that causes cells that initially internalize particles to then more actively search for new particles.

One important goal of our study is to develop a set of design parameters by which we can better deliver particles to macrophages for specific biomedical applications. It may be useful to target the microparticles to a subset of macrophages by defining a set of physical and biochemical signals through a combination of particle size, avidity, and/or other (as yet untested) receptor ligands for “designer drug” therapies. We found the percentage of phagocytic cells is dependent on both particle size and Fc density. The maximum number of particles per cell were internalized for the smaller 0.5 µm and 1 µm particles functionalized with high Fc densities, despite only approximately 65% of the cells classified as phagocytic. The mid-sized particles of 2 µm are phagocytosed by the highest percentage of macrophages (90%), regardless of Fc density. The larger 3 µm and 4.5 µm particles, while only phagocytosed by a smaller portion of the macrophage sample, nonetheless accounted for the greatest amount of total volume of microparticles phagocytosed.

Our attachment data shows that the initial interaction and attachment of the microparticle with the macrophage was most strongly affected by particle size. However, phagocytic activity and particle internalization was found to be dependent on both particle size and Fc density. The kinetics of internalization may also influence the phagocytic activity of macrophages. A previous study investigated the internalization rates of particles from 3–9 µm and found no significant difference in the phagocytic rate for the sizes tested, even when particles were functionalized with Fc. However, internalization rates of smaller particles and the effect of Fc density were not evaluated [25].

A possible explanation for a significant dependence on size in the initial and ongoing processes of phagocytosis has been posited to result from the dynamics of cell signaling components recruited in the process. A theoretical model suggests that larger particles will require the cell membrane receptors to diffuse over larger distances resulting in the cell membrane taking longer to envelop the particle. Particle size can also lead stalling of the phagocytosis process and lead to the low phagocytosis rates we found for the 3 µm and 4.5 µm particles [31]. Smaller particles require the recruitment of fewer signaling components before their engulfment, providing for less time to be affected by inhibitory signals such as wortmannin, LY294002, or dominant-negative SHIP-1 [40], [41]. Furthermore, it has been suggested that there is a specific size threshold of 0.5–1 µm whereby the macrophage phagocytosis transitions from a coated vesicle-mediated uptake into an actin-mediated uptake. While actin is crucial for the formation of the phagocytic cup, these reports suggest a size threshold of 2 µm before inhibitors that prevent Fc receptor signaling to the actin cytoskeleton are generated [9], [40], [41]. The action of size-dependent inhibitory signals would also account for why there is a consistently low average number of microparticles per cell for the 3 µm and 4.5 µm particles regardless of Fc density. It will be important to more fully understand these inhibitors if we hope to take advantage of the increased volume of delivery associated with internalization of larger particles. For example, nanoparticles are frequently touted as superior delivery method due to their greater surface area per unit volume compared to microparticles [42]. However, the volume of particles decreases with the third power of diameter, which is a detriment for delivering significant amounts of encapsulated drugs or imaging agents into the cell interior via nanoparticles.

Not only are these results relevant to the direct delivery of microparticles to macrophages, but they are also important to understanding the downstream effects of the internalization of these particles. The recognition of immune threats can occur through physical cues or the many different biological receptors on the cell surface. A major distinction between the different receptor pathways is the immune-activation state they induce in the macrophage. Receptors that bind to “self” products intended for waste removal will not trigger immune activation; those cells will simply increase in size (if necessary) and then engulf and degrade the recognized substance in a non-inflammatory process. However, receptors that recognize substances that are potentially dangerous to the body (e.g. through PAMP-receptors or Fc receptors) will trigger activation of the macrophage, leading to secretion of immune mediators and enhanced intracellular degradative capacity to deal with the active insult represented by the engulfed threat. Opsonization of microparticles tends to favor activation of cells as it is a natural mechanism of targeting foreign substances for recognition and response, but will tend to not lead to full cellular activation in the absence of secondary “danger signals” [9], [33]. In the future we plan to examine how the size and Fc density of particles also affect cytokine production during and after phagocytosis, indicating changes in macrophage phenotype. As we investigate the activation of the immune system with Fc-functionalized particles, the inclusion of bioactive substances such as small-molecule drugs, antibiotics, cytokines or chemokines, or vaccine targets, either on or contained in the microparticles, could lead to a broad-based immune delivery system that is tunable to experimental or clinical needs: one that could be either inflammatory or not; affect a broad or more targeted population of macrophage cells; or deliver small amounts of highly active substances, or larger amounts of less active materials.

## Supporting Information

Figure S1 **Overview of the phagocytosis assay.** (A) RAW 264.7 murine macrophage cells are plated at low density (B) Cells are allowed to incubate, grow, and spread for ∼36 hrs (C) Wells are washed and replaced with serum free media before beads are added (D) Beads are added and allowed to incubate for ∼1.5 hrs (E) Wells are washed at least 3 times and replaced with PBS (F) Wells are scraped and contents transferred to a microcentrifuge tube for Flow Cytometry.
(TIF)

Figure S2 **Amounts of BSA and anti-BSA IgG added for each particle size and Fc density ratio condition.**
(TIF)

Figure S3 **Normalized mean fluorescence intensity of yellow-green fluorescent microparticles labeled with TexasRed fluorescently conjugated secondary antibodies.** Decreasing normalized MFI corresponds to decreased Fc density for fluorescent particles used in flow cytometry experiments.
(TIF)

Figure S4 **Surface area of particles internalized per cell for different functionalizations.** The total surface area of internalized particles by macrophages for different particle sizes and Fc densities. BSA-only particles are shown for comparison.
(TIF)

Figure S5 **Volume of particles internalized per cell for different functionalizations.** The total volume of internalized particles by macrophages for different particle sizes and Fc densities. BSA-only particles are shown for comparison.
(TIF)
