# Supplementary figures and images for: Effects of Microparticle Size and Fc Density on Macrophage Phagocytosis
Source: PLoS One. 2013 Apr 22;8(4):e60989. doi: 10.1371/journal.pone.0060989 (PMC3632606; doi:10.1371/journal.pone.0060989)

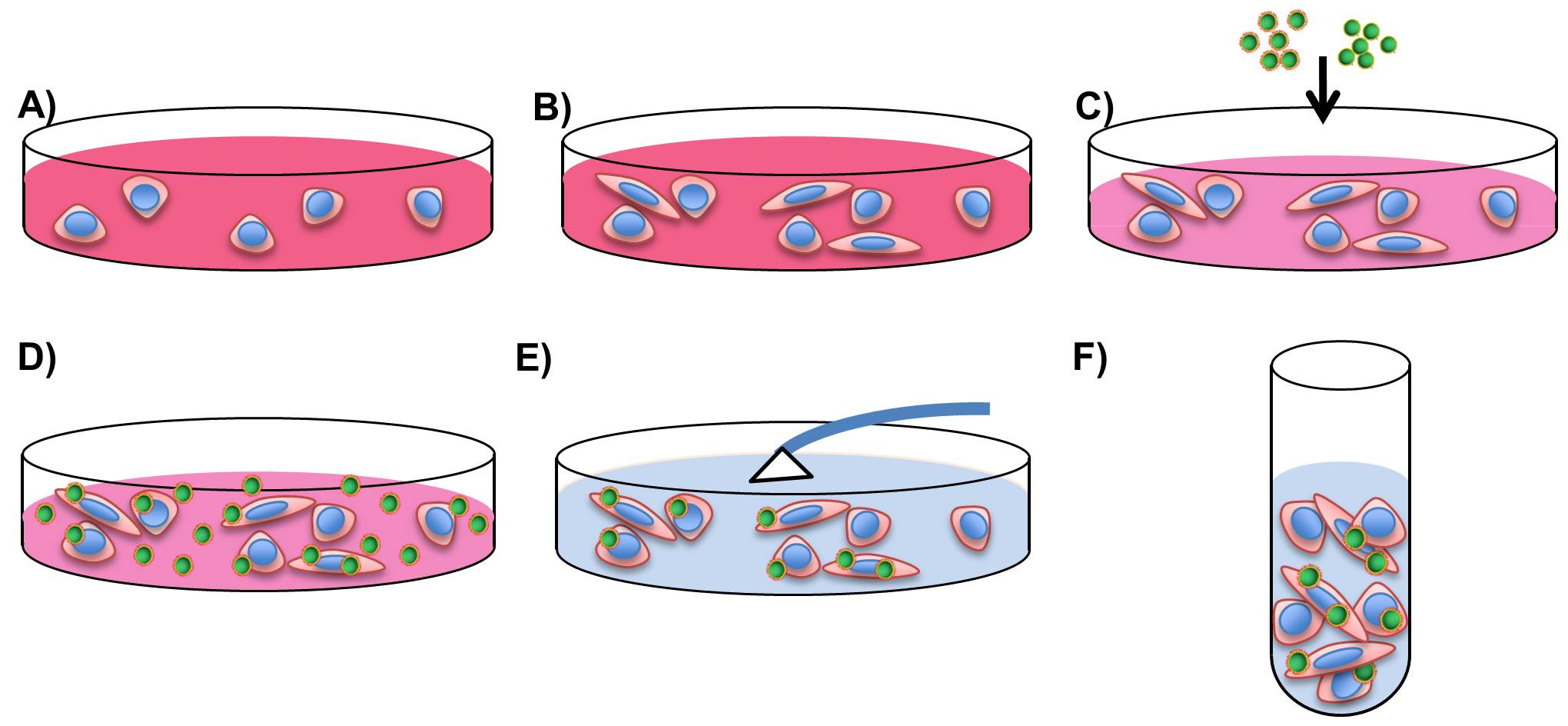

Supplement: Figure S1 — Overview of the phagocytosis assay. (A) RAW 264.7 murine macrophage cells are plated at low density (B) Cells are allowed to incubate, grow, and spread for ∼36 hrs (C) Wells are washed and replaced with serum free media before beads are added (D) Beads are added and allowed to incubate for ∼1.5 hrs (E) Wells are washed at least 3 times and replaced with PBS (F) Wells are scraped and contents transferred to a microcentrifuge tube for Flow Cytometry. (TIF) [file pone.0060989.s001.tif]

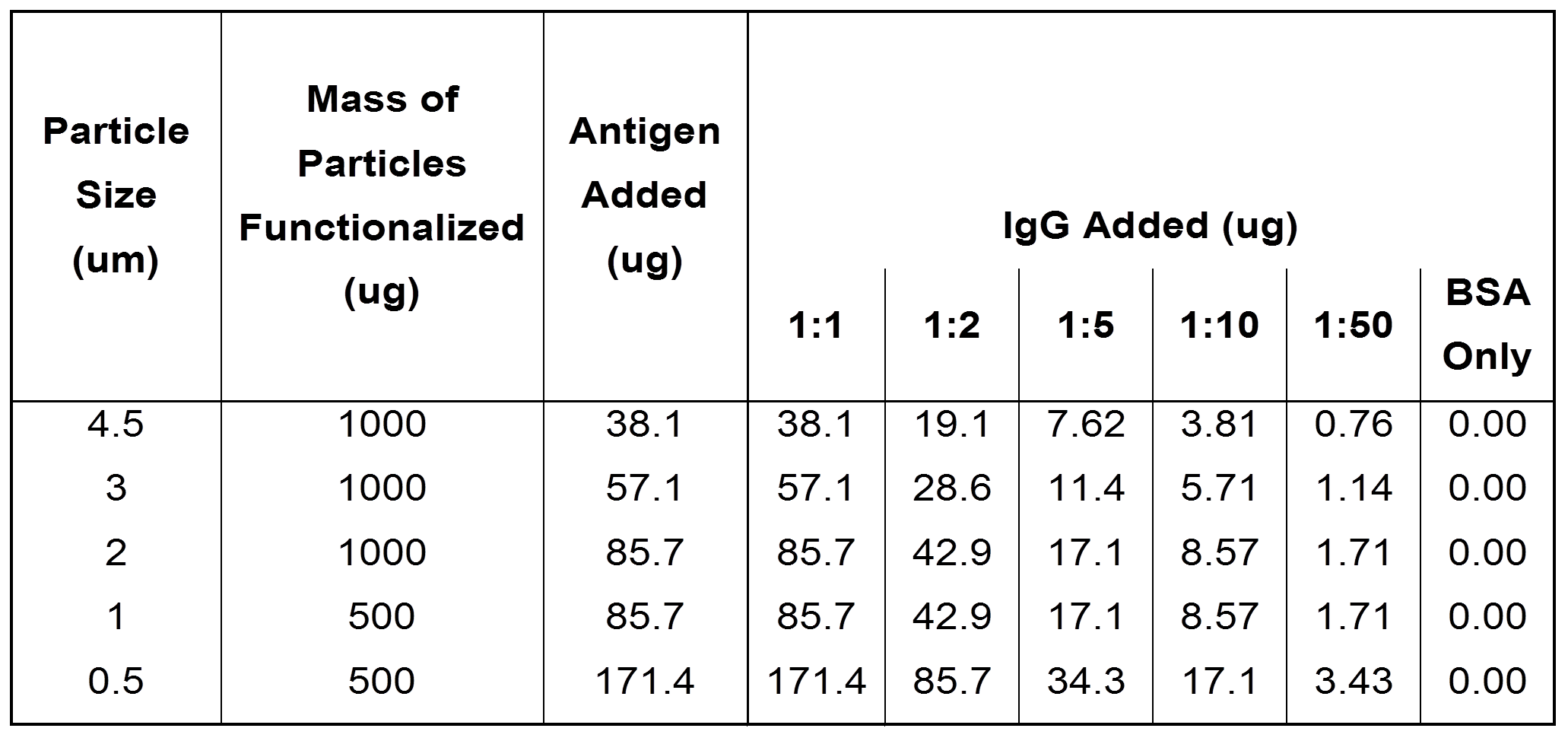

Supplement: Figure S2 — Amounts of BSA and anti-BSA IgG added for each particle size and Fc density ratio condition. (TIF) [file pone.0060989.s002.tif]

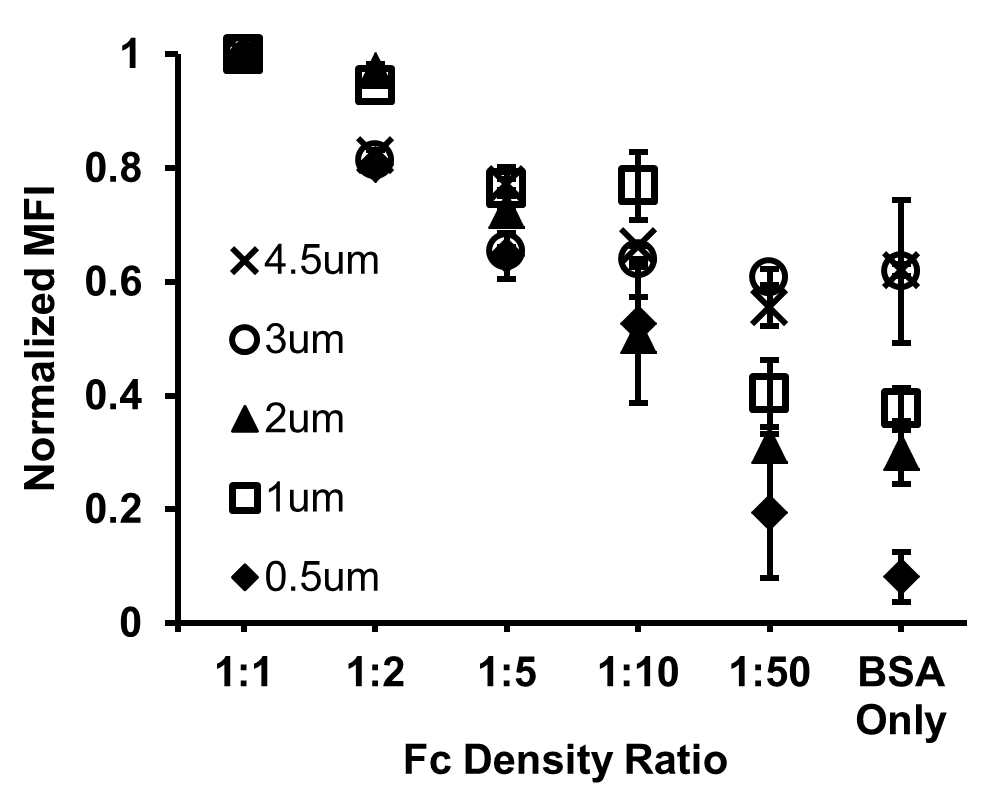

Supplement: Figure S3 — Normalized mean fluorescence intensity of yellow-green fluorescent microparticles labeled with TexasRed fluorescently conjugated secondary antibodies. Decreasing normalized MFI corresponds to decreased Fc density for fluorescent particles used in flow cytometry experiments. (TIF) [file pone.0060989.s003.tif]

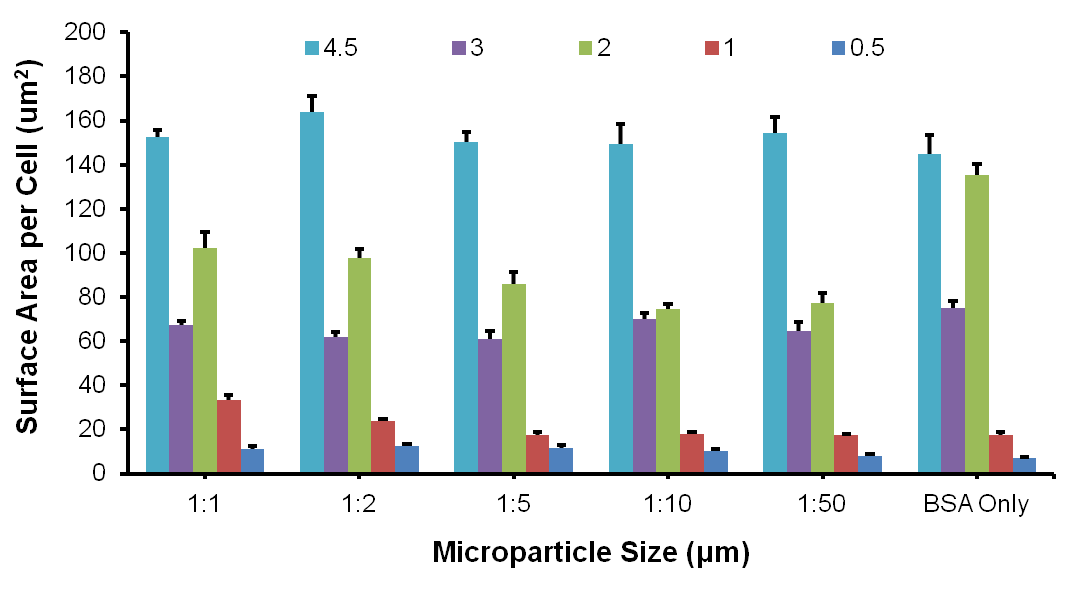

Supplement: Figure S4 — Surface area of particles internalized per cell for different functionalizations. The total surface area of internalized particles by macrophages for different particle sizes and Fc densities. BSA-only particles are shown for comparison. (TIF) [file pone.0060989.s004.tif]

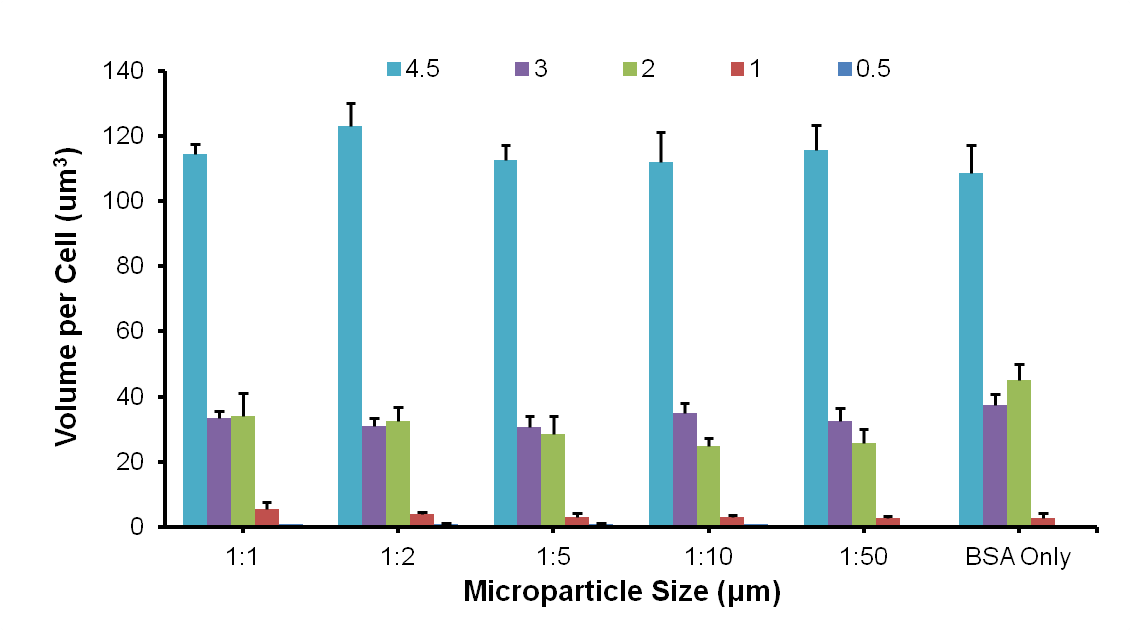

Supplement: Figure S5 — Volume of particles internalized per cell for different functionalizations. The total volume of internalized particles by macrophages for different particle sizes and Fc densities. BSA-only particles are shown for comparison. (TIF) [file pone.0060989.s005.tif]
